# Gender Difference in Bacteria Endotoxin-Induced Inflammatory and Anorexic Responses

**DOI:** 10.1371/journal.pone.0162971

**Published:** 2016-09-15

**Authors:** Shiu-Ming Kuo

**Affiliations:** Department of Exercise and Nutrition Sciences, University at Buffalo, Buffalo, New York, United States of America; Medical University of Vienna, AUSTRIA

## Abstract

Inflammation-related anorexic response has been observed in systemic diseases as well as in localized infection and is an important issue in patient care. We tested the hypothesis that upon the same endotoxin exposure, males have more severe inflammatory responses and thus suffer from more negative effect on appetite. Ten-week old male and female mice were compared in their plasma levels of pro-inflammatory cytokines after a body weight-based *i*.*p*. injection of bacterial endotoxin lipopolysaccharide. Male mice consistently showed significantly higher levels of IL6 and TNFα than female mice. The difference was observed starting at 3 hours after the systemic endotoxin exposure. It was independent of the level of endotoxin dosage and of the genotype of the anti-inflammatory cytokine, IL10. Interestingly, endotoxin-injected male mice also had significantly higher plasma IL10 levels compared to the female mice. Pre-puberty young mice showed no gender differences in the plasma levels of IL6, TNFα and IL10. Their cytokine levels were mostly between that of the adult males and females. Consistent with the higher inflammatory response in male mice, the endotoxin exposure also led to significantly more appetite loss in male mice at a range of doses in two strains of mice. Saline injection in the absence of endotoxin affected neither the cytokine levels nor the appetite. Although a direct mechanistic link between inflammation parameters and appetite was not addressed here, the results support that male gender could be a risk factor for higher pro-inflammatory cytokines and anorexic response after the endotoxin exposure.

## Introduction

Bacterial endotoxin exposure is known to induce defensive innate immune response including the secretion of pro- and anti-inflammatory cytokines. While the purpose of the innate immune response is for protection, the immune response is also known to be the cause of various sickness behavior such as a reduced intake of food and beverage [1, 2]. Since the anorexic response was observed widely in acute and chronic diseases [3–5], it is a significant concern in disease management and clinical nutrition [3, 4].

Cytokines were indicated as the mediator of the sickness behavior. Cytokines and their receptors are found in the brain [6]. Increased levels of pro-inflammatory cytokine TNFα, either by a direct central TNFα injection or as a consequence of endotoxin exposure, led to sickness behavior in a dose- and time-dependent fashion [7, 8]. Anti-TNFα antibody therapy, on the other hand, is associated with an increase in body weight [9]. Pro-inflammatory cytokine IL6 also promoted the sickness response as confirmed through experiments using IL6-knockout mice [10] or applying an inhibitor of IL6 signaling [11]. IL6-knockout and the presence of IL6 inhibitor both reduced the severity of sickness behavior. The balance of pro- and anti-inflammatory cytokines is critical in the physiological response to endotoxin and the secretion of anti-inflammatory cytokine IL10 was also affected during illness [12]. IL10-knockout led to an exaggerated response to endotoxin [13] including profound fever and more death at high dose [14]. Centrally administered IL10 can inhibit sickness behavior whereas centrally administered monoclonal antibody against IL10 can promote the sickness behavior [15, 16]. In addition, human IL10 gene polymorphism increased sickness response [17].

A gender difference in the endotoxin and pathogen-induced inflammation has been reported in adult humans and animals. However, the conclusions were not all consistent maybe due to a difference in the health condition of the study subjects. Because the sickness behavior also involves the neurocircuits in the brainstem [5, 18, 19], it is also not clear whether a difference in the inflammation alone can explain the gender behavior difference. In some studies, more pro-inflammatory innate immune responses were reported in female subjects [20, 21] and female rodents [22, 23]. Others found instead that male gender is a risk factor for more inflammation-associated physiological changes upon exposure. After an intravenous injection of lipopolysaccharide (LPS) endotoxin, men (aged 29±1 years) showed higher fever than women (aged 26±1 years) but their inflammation-related cytokine levels were not different [24]. *Ex vivo* studies, however, found a higher level of endotoxin-induced TNFα and IL10 synthesis by the blood samples from male subjects [25, 26]. Male mice showed greater airway hyper-responsiveness than females after intratracheal endotoxin administration along with a higher level of TNFα [27]. Human viral hepatitis and murine pathogen-induced hepatocellular carcinoma were both more prevalent in males. A higher pro-inflammatory TNFα was observed in males in the mouse model of liver disease [28]. Male mice also had higher serum IL6 levels compared to females after chemical-induced liver injury [29]. Plasma IL6 and mortality were both found to be higher in male septic patients compared to the female patients of similar age [30]. A possible gender difference in the anorexic response during inflammation was not studied.

The goals of this study are to determine the effect of gender on the severity of the anorexic behavior and whether a gender difference in the inflammatory cytokines can explain the difference in behavior. To investigate the effect of gender on the anorexic response during inflammation, mice raised in the specific pathogen-free facility were used. Stress was shown to affect the immune response [31]. To avoid the confounding effect of stress, mice were only handled for genotyping and weekly cage change. Because of the inconsistent gender effects reported in previous publications as summarized above, the possible dose- and time-dependent gender differences were also considered and a range of LPS doses and time points were used here. The levels of pro- and anti-inflammatory cytokines (TNFα, IL6, IL10) were determined in adult and pre-puberty young mice of both genders to examine the effect of sex hormones. In addition, wildtype, IL10+/- and IL10-null mice were also compared to determine the contribution of IL10 in the gender difference in the inflammatory response. To be able to generate a broader conclusion, the correlation between the level of plasma pro-inflammatory cytokine and anorexic response was examined in two strains of mice.

## Materials and Methods

### Animal care

Protocols for mouse colony maintenance and mouse handling were approved by the Institutional Animal Care and Use Committee of the University at Buffalo. Male and female wildtype, IL10+/-, and IL10-/- mice were from an IL10+/- colony maintained in the UB specific pathogen-free facility as described before [32]. They were given nutritionally balanced commercial NIH31 diet. The colony was propagated by heterozygous mating with PCR genotyping and no clinical or histological abnormalities were observed in IL10-null mice up to 10 weeks of age under our housing conditions [32, 33]. IL10 null mice have no detectable IL10 expression. 129S6 mice were from a separately maintained colony as reported before and were given nutritionally balanced commercial Picolab Mouse Diet 20 [34]. Mice were individually housed in plastic cages with elevated food tray to enable individual food consumption measurement.

### Endotoxin injection

Stock endotoxin LPS (from Escherichia coli 0127:B8, catalog number L-3880, 1x10^6^ endotoxin unit (EU)/mg, Sigma-Aldrich) solutions of several concentrations were prepared in sterile saline. They were given through *i*.*p*. injection from 20 EU/g (= 0.02 μg/(g body weight·5 μL saline)) to 5,000 EU/g (= 5 μg/(g body weight·5 μL saline)). Based on the observations from our preliminary studies and publication [35], this range of LPS dosage was chosen to induce mild to severe but not lethal inflammation. Body weight-based LPS dosage is generally used in similar studies [20, 21, 23, 24]. The dose used for each experiment was described in the Figure and Table legends. LPS and saline injections were routinely performed in the middle of the 12-hr light cycle after a brief alcohol wiping of the injection area. To ensure that there was no injection injury, signs of internal bleeding were checked during the needle retrieval. No injection injury was observed in the entire study. The alertness of injected mice was monitored hourly by non-intrusive gentle cage-tapping during the remaining 6 hours of the light cycle or until they were killed. Except in IL10-/- mouse studies where fatality measurement was the end point, no mortality was observed prior to the experimental endpoints.

### Plasma collection and Cytokine measurements

All mice were killed at the designated time points after the injection by cervical dislocation as described in the Figures and Table. Trunk blood was collected after decapitation. Plasma was separated from the blood cells by centrifuging heparinized blood at 1,000 xg for 10 minutes and then stored in -80°C freezer until cytokine measurements. The plasma levels of IL6, TNFα, and IL10 were measured by commercially available specific ELISA kits following the manufacturers' instructions. Each 96-well plate of measurement included a cytokine standard curve. The log-linear detection ranges were 4–500 pg/mL for IL6 (eBioscience, #88–7064), 8–1000 pg/mL for TNFα (eBioscience, #88–7324), and 4–1000 pg/mL for IL10 (R&D Systems, #M1000). To ensure that plasma OD_450_ readings were within the range of the OD_450_ readings of the standards on the plate, plasma samples from LPS-injected mice mostly required proper dilutions (at up to 8,000 fold). For the quantification of all three cytokines, the OD_450_ measurement of a plasma-free blank was subtracted from sample OD_450_ measurements before calculating the plasma cytokine concentrations.

### Statistical analysis

**Fig 1:**
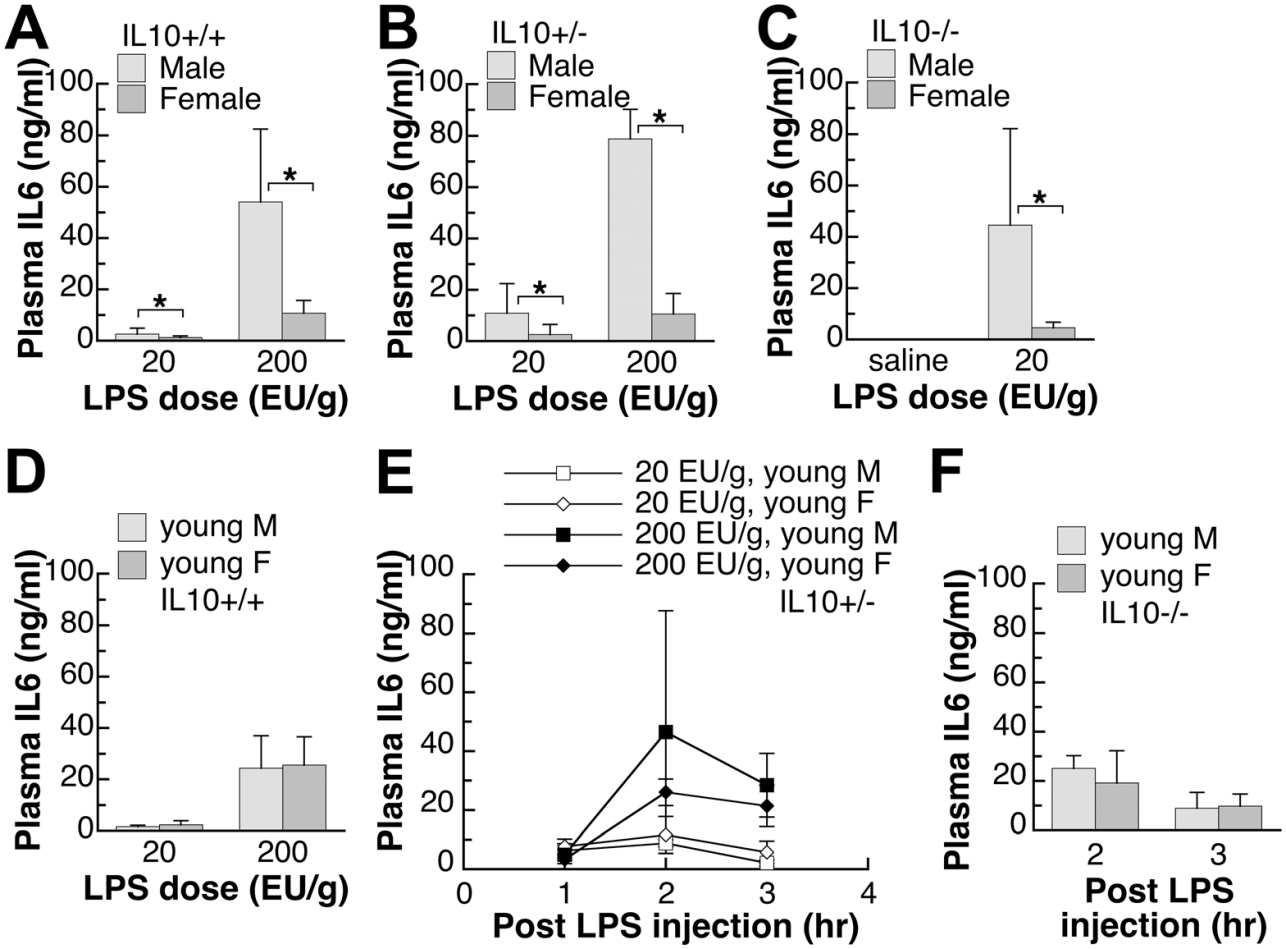
Plasma IL6 levels of endotoxin-injected male and female mice with different IL10 genotypes. (A,B,C) The adult mice were 9–10 weeks old and the IL6 levels were determined in plasma samples collected at three hours after injection. Saline injection did not increase the plasma IL6 levels to above that of the non-injected mice in either gender. (D,E,F) Young mice were 5–6 weeks old and young IL10-/- mice were injected with endotoxin at 20 EU/g. Different sets of mice were used for experiments at different time points. IL6 plasma values shown are mean±SD of *n* = 5–8 mice in each group. Student’s *t*-test was used to determine the effect of gender. *: significantly different at p<0.05.

**Fig 2:**
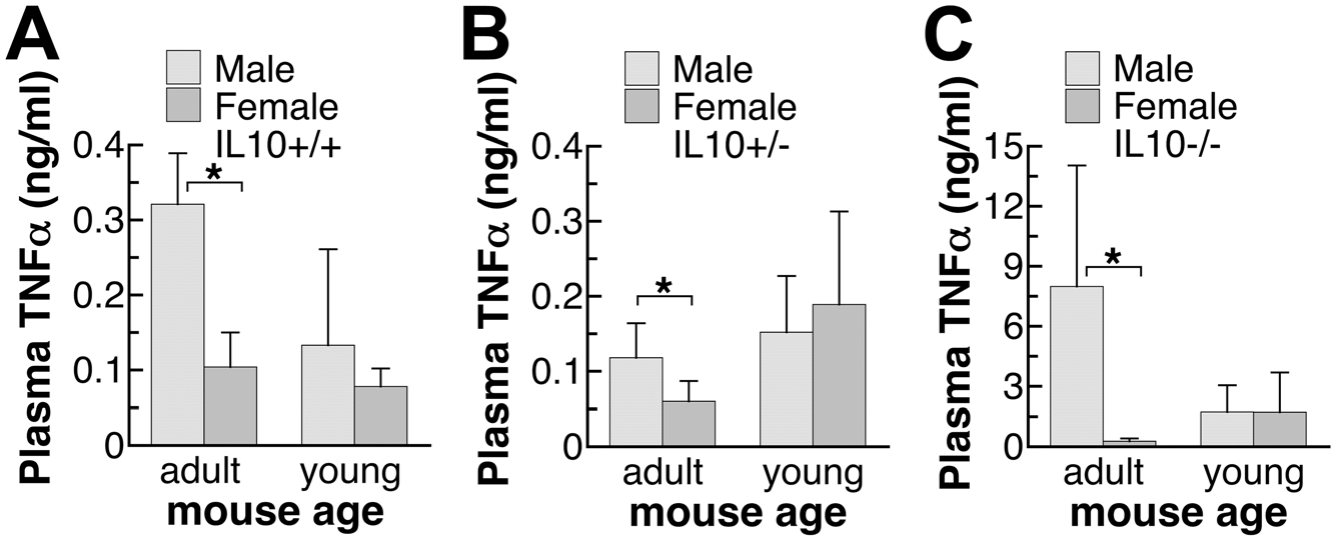
Plasma TNFα levels of endotoxin-injected male and female mice with different IL10 genotypes. (A,B,C) The adult mice were 9–10 weeks old and young mice were 5–6 weeks old. The TNFα levels were determined in plasma samples collected at three hours after the injection of endotoxin, LPS, at 20 EU/g. TNFα plasma values shown are mean±SD of *n* = 5–8 mice in each group. Student’s *t*-test was used to determine the effect of gender. *: significantly different at p<0.05.

**Fig 3:**
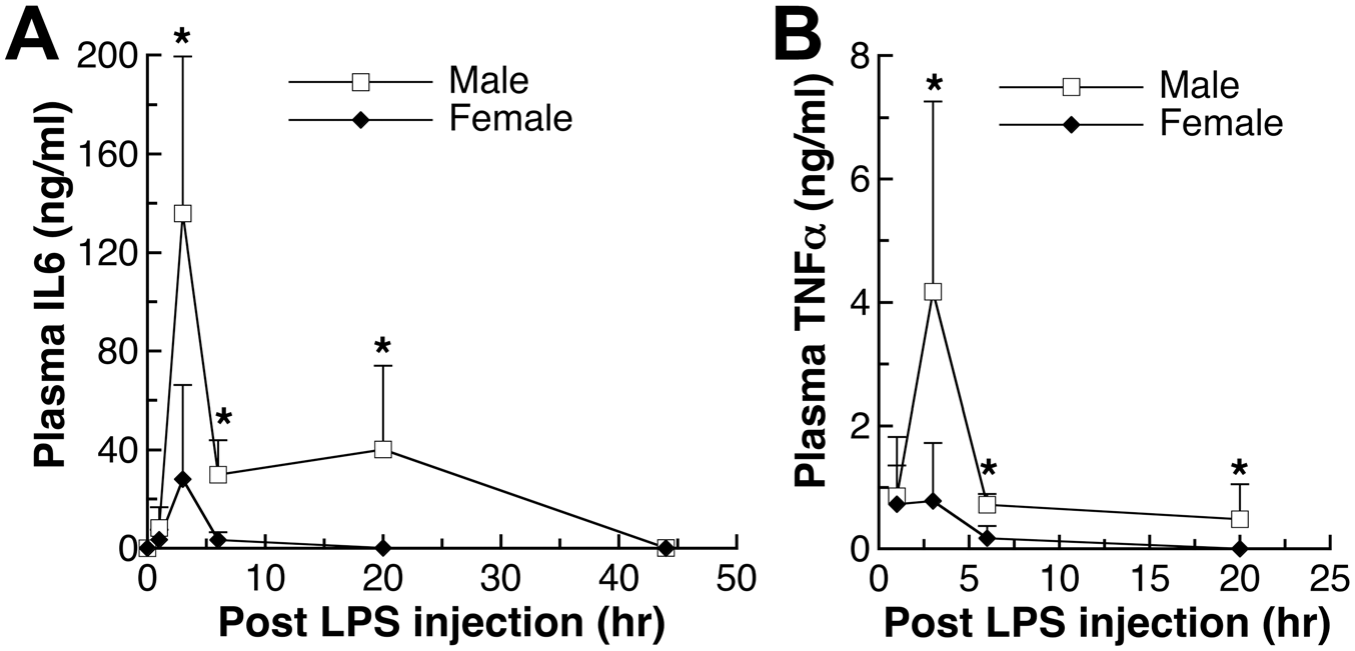
Time-dependent changes of plasma IL6 and TNFα levels after endotoxin-injection in adult male and female mice. IL10-/- mice at 9–10 weeks old were given LPS injection at 100 EU/kg body weight. Different sets of mice were used for experiments at different time points. (A) IL6 and (B) TNFα plasma values shown are mean±SD of *n* = 4–6 mice in each group. Student’s *t*-test was used to determine the effect of gender. *: significantly different at p<0.05.

**Fig 4:**
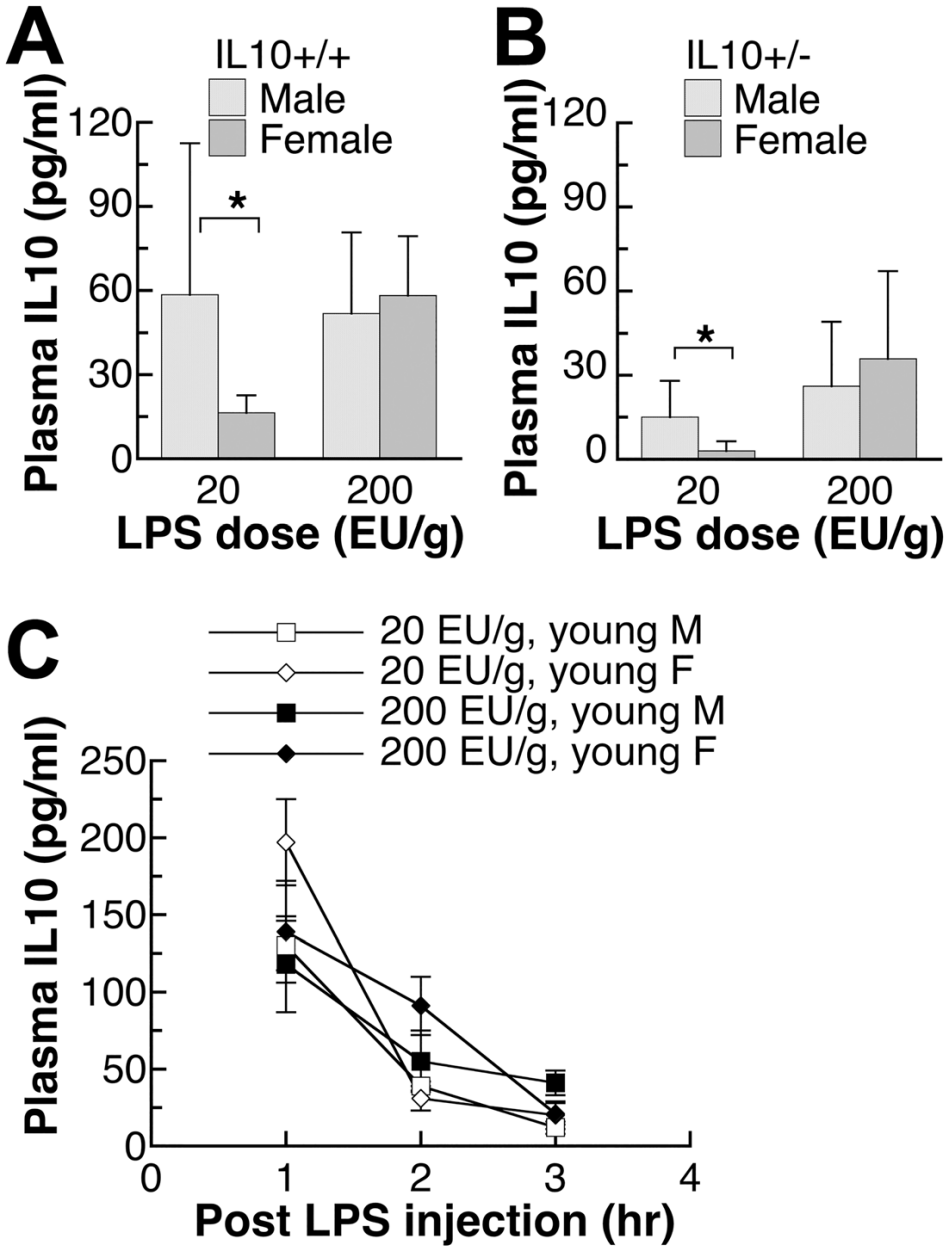
Plasma IL10 levels of endotoxin-injected male and female mice with IL10+/+ or IL10+/- genotypes. (A,B) The adult IL10+/+ or IL10+/- mice were 9–10 weeks old. IL10 levels were determined in plasma samples collected at three hours after LPS injection at two different dosages of LPS. (C) The young IL10+/- mice were 5–6 weeks old and their plasma levels were collected at 1, 2, 3 hours after two different LPS dosages. Different sets of mice were used for experiments at different time points. IL10 plasma values shown are mean±SD of *n* = 4–7 mice in each group. Student’s *t*-test was used to determine the effect of gender. *: significantly different at p<0.05.

**Fig 5:**
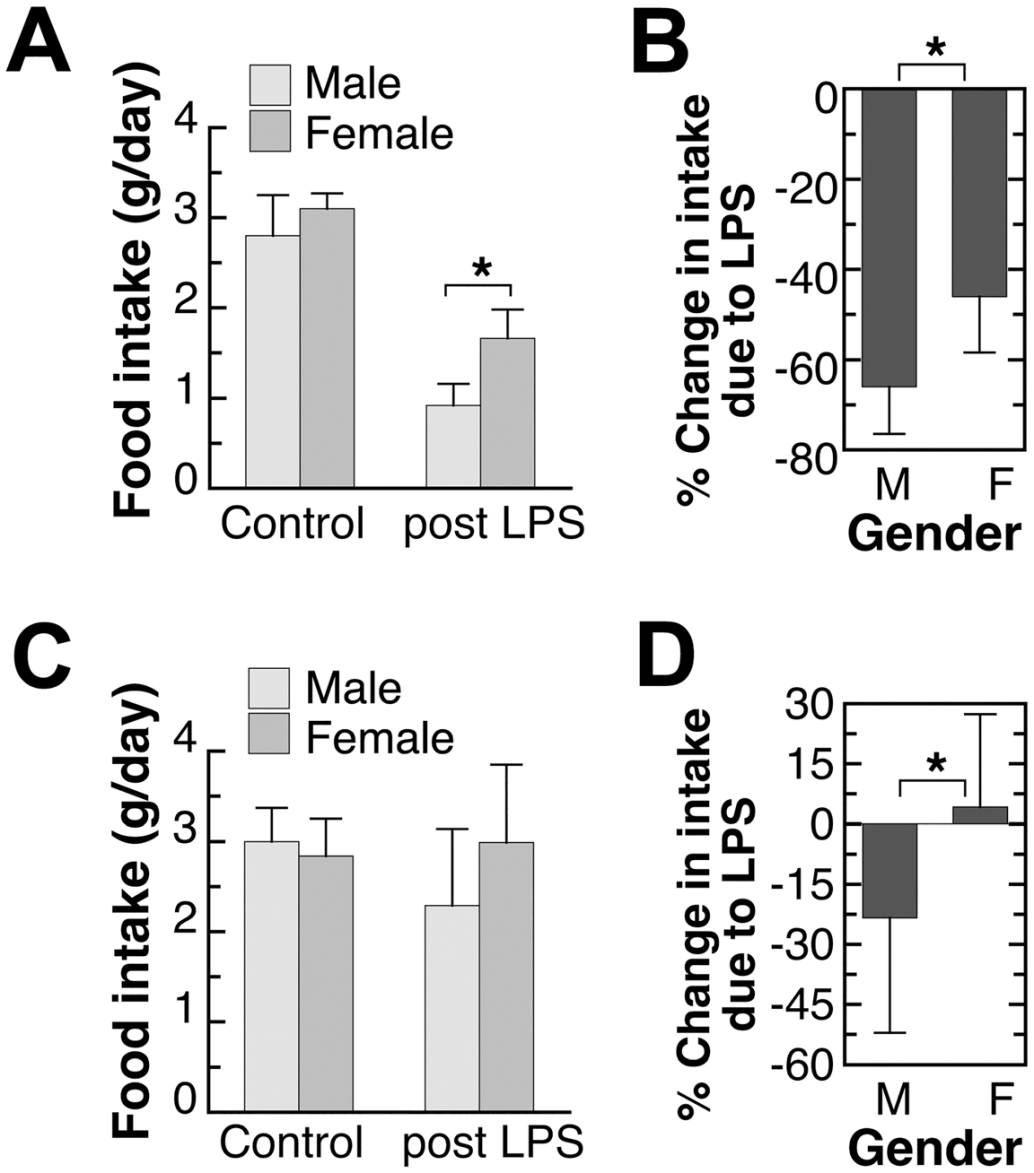
Effect of endotoxin-exposure on 24-hour food intake of adult male and female mice. The adult mice were 9–10 weeks old. The control daily food intake was the average daily food intake during the week prior to the endotoxin injection (non-injected mice). The post LPS intake was the 24-hour intake after the endotoxin injection. The % change was determined by the difference between the control intake and post LPS intake of individual mouse. Results shown in (A) and (B) were from different sets of mice given (A) 1,000 EU endotoxin/g body weight or (B) 200 EU endotoxin/g body weight. Values shown are mean±SD of *n* = 5–8 mice in each group. Student’s *t*-test was used to determine the effect of gender. *: significantly different at p<0.05.

All values are shown as mean±SD and a significant effect is defined as *P*<0.05. Following the main focus on gender difference in Figs 1, 2, 3, 4 and 5, Student’s *t* test was used to determine the effect of gender under each LPS dose and time point. In Table 1, 2-way ANOVA was used to determine the effects and interaction of gender and injection. Because of a significant interaction between gender and injection, the main effects, gender and injection, cannot be concluded and thus routine post-hoc analysis was not used. Instead, the nonparametric Mann-Whitney U test was used to further determine the effect of gender under each individual condition.

**Table 1:** Food intake and plasma IL6 levels of adult male and female 129S6 mice injected with saline or endotoxin in saline^a^.

	Injection	*n*^b^	Intake before injection	Intake after injection	Fractional change in intake	Plasma IL6 (24hr after injection)
			*g/day*	*g/day*	*%*	*pg/ml*
Male	Saline^c^	12	3.30±0.84	4.10±0.82	27.7±27.7	0.50±0.73
	LPS^c^	12	3.13±0.50	1.02±0.87	-67.0±28.6	114±46
Female	Saline^c^	15	2.91±0.26	3.28±0.75	13.4±25.9	0.63±1.81
	LPS^c^	12	2.88±0.30	1.56±0.62	-46.8±17.2	52±19
			*P-value*^d^			
Gender			0.030	0.512	0.678	0.0001
Injection			0.506	0.0001	0.0001	0.0001
Interaction			0.614	0.0027	0.019	0.0001

^a^Values are means ± SD.

^b^Number of mice in each group. Adult mice were used for experiment at 9–10 weeks of age.

^c^Endotoxin lipopolysaccharide was injected *i*.*p*. at 5,000 EU/g body weight with saline as the vehicle at 5 μL saline per g body weight. The saline group received saline at 5 μL saline per g body weight.

^d^Two-way ANOVA was used for the data analysis.

## Results

### Plasma IL6 levels after endotoxin injection

#### Adult mice

To characterize the effect of gender on the response to endotoxin, plasma levels of pro-inflammatory cytokine, IL6, were measured in male and female mice (Fig 1). In adult wild-type mice (Fig 1A), higher LPS exposure led to higher plasma IL6 levels at 3 hours after the injection and male mice had significantly higher IL6 levels at both LPS doses than female mice. To determine whether the expression of anti-inflammatory cytokine, IL10, affects the gender-difference in IL6 response, similar experiments were also carried out in adult IL10+/- mice (Fig 1B) and adult IL10-/- mice (Fig 1C). The loss of IL10 led to higher IL6 levels under the same level of LPS exposure (Fig 1A vs. 1B vs. 1C) but adult male mice consistently showed significantly higher IL6 levels compared to the adult female mice of the same genotype. Because of a complete lack of the anti-inflammatory cytokine IL10, IL10-/- mice were only used for the 20 EU/g LPS dose. In two preliminary experiments of 200 EU/g dose, adult male IL10-/- mice suffered from fatality (3/7 and 2/7) but none of the female IL10-/- mice died from the same dose (0/9 and 0/7). Plasma IL6 levels were generally less than 10^−2^ ng/ml after the vehicle saline injection and were similar between male and female mice (Fig 1C). Non-injected mice, saline-injected mice of other genotypes, and mice at 44–116 hours after LPS injection also had similarly minimal plasma levels of IL6 (results not shown).

#### Pre-puberty mice

Non-intervened pre-puberty young male and female mice were used as a model to examine the importance of sex hormones in the higher male IL6 levels observed in Fig 1A, 1B and 1C. In parallel to the adult mice experiment in Fig 1A, 1B and 1C, pre-puberty young male and female mice of all three genotypes, IL10+/+, IL10+/- and IL10-/-, were all studied. Similar to adult mice, higher LPS dose led to higher plasma levels of IL6 in young mice (Fig 1D and 1E). The loss of IL10 also led to higher IL6 levels under the same level of LPS exposure in young mice (Fig 1D vs. 1E vs. 1F). Because of the exaggerated inflammatory response, the young IL10-null mice were similarly only used for 20 EU/g LPS dosage (Fig 1F). Different from the adult mice, there was no gender effect on plasma IL6 levels in any genotype of pre-pubertal mice at any doses of LPS or time points (Fig 1D, 1E and 1F).

### Plasma TNFα levels in adult and pre-pubertal mice

Plasma levels of another pro-inflammatory cytokine, TNFα, were also measured (Fig 2A, 2B and 2C). Similar to IL6 observations of adult mice in Fig 1A, 1B and 1C, adult male mice had significantly higher TNFα levels in response to endotoxin LPS challenge compared to adult female mice. Mice of three genotypes, IL10+/+, IL10+/- and IL10-/-, showed similar gender effects. IL10-null mice, under the exaggerated inflammatory response, showed especially large gender difference (Fig 2C vs. 2A). This gender difference, however, was not observed in pre-puberty young mice (Fig 2A, 2B and 2C).

### Time-dependent changes in IL6 and TNFα levels in adult mice

To determine whether adult males have higher pro-inflammatory cytokine levels throughout the response to LPS, we conducted time-dependent studies following the plasma IL6 and TNFα levels after LPS injection and the results are shown in Fig 3. There was no gender difference at 1 hour after the LPS injection (Fig 3A and 3B). At the peak cytokine levels, 3 hours after the injection, the gender difference of both IL6 and TNFα became evident. The significantly higher levels in adult male mice persisted for both cytokines as the cytokine levels gradually declined to the basal level (Fig 3A and 3B).

### Plasma IL10 levels after endotoxin injection

Comparing the results in Figs 1 and 2 among mice of three IL10 genotypes, it is clear that the absence of IL10, while affected the innate immune response to endotoxin, did not affect the gender difference. Little information is available on the effect gender on the plasma IL10 levels after LPS exposure. Only wildtype and IL10+/- mice were used in Fig 4 because IL10-null mice have no IL10 expression. As shown in Fig 4A and 4B, adult males also had significantly higher levels of IL10 at 3 hours after the injection of 20 EU/g LPS. This was true for both IL10+/+ and IL10+/- genotypes. At higher dose of 200 EU/g, there were no gender differences in the level of IL10. In young mice (Fig 4C), neither LPS dosage led to a gender difference in the plasma level of IL10 at three time points, 1,2, 3 hours after the injection.

### Anorexic response after endotoxin injection

#### IL10+/- colony

The anorexic response of adult mice after the LPS injection was determined by measuring 24-hour food intake and the results are shown in Fig 5. Food intake was shown both as intake per day (Fig 5A and 5C) and % change in intake after LPS injection (Fig 5B and 5D). Food intake before LPS injection, represents the intake of non-injected adult mice, was similar between two studies and comparable between male and female (the left set of bars in Fig 5A and 5C). Higher dosage of endotoxin led to more severe anorexic response (Fig 5A and 5C vs. 5B and 5D). Nevertheless, at both levels of LPS injection, the anorexic response was significantly more pronounced in males. During the first 24 hours after the LPS injection, male mice had more loss of appetite compared to females (Fig 5B and 5D).

#### 129S6 mice

A previous study reported an effect of mouse genetic background on their gender difference in the inflammatory response [22]. The mice of three IL10 genotypes that we reported in Figs 1, 2, 3, 4 and 5 shared the same genetic background. In Table 1, the food intake and plasma IL6 levels after LPS injection were determined in male and female mice of a different genetic background. In Table 1, the effect of injection was also determined by including a separate set of mice given saline (vehicle) injection using a two-way experimental design. Before injection, gender had a significant effect on food intake with male mice consuming significantly more food (Table 1, the 1^st^ data column). After the injection, a significant gender-injection interaction on food intake was observed (Table 1, the 2^nd^ data column). This interaction was also significant when the data was expressed as fractional changes in intake (Table 1, the 3^rd^ data column). Because of the interaction, a main effect of gender cannot be concluded. The nonparametric Mann-Whitney U test for results in the 2^nd^ and the 3^rd^ data columns revealed that the gender effect depended on the type of injection and thus explained an interaction in the 2-way ANOVA. While the saline-injected male mice consumed significantly more food than the saline- injected female mice similar to the period prior to the injection (Table 1, the 1^st^ data column), the LPS-injected male mice consumed significantly less food than the LPS-injected female mice (p<0.05). The fractional change in food intake was not different between saline-injected male and female mice while the LPS-injected male mice had significantly more decrease in food intake compared to the LPS-injected female mice (p<0.05). Student’s *t* test performed on results in the 2^nd^ and the 3^rd^ data columns to examine the effect of gender led to the same conclusion: more loss of appetite in male mice compared to female mice after the LPS injection.

#### IL6 and anorexic response

A significant gender-injection interaction in the IL6 levels was also observed in these mice (Table 1, the 4^th^ data column), which implies a role of injection in the gender difference. To determine the gender effect under each injection condition, the nonparametric Mann-Whitney U test was used. The test revealed that male mice had significantly higher plasma IL6 levels at 24-hour after the LPS injection compared to the female mice (p<0.05) but there was no gender difference in the plasma IL6 levels of saline-injected mice. Student’s *t* test performed on the IL6 results led to the same conclusion: higher IL6 in male mice compared to female mice only after the LPS injection.

## Discussion

Based on the series of experiments presented here on healthy specific pathogen-free and minimally intervened mice, males appear to have a stronger innate immune response, when challenged with a body weight-based dose of endotoxin. Some other laboratories also reported higher plasma levels of pro-inflammatory cytokines TNFα in male mice upon the exposure to endotoxin [27]. While the absence of IL10 was shown to lead to exaggerated pro-inflammatory response (Figs 1C and 2C) and eventual death in our preliminary study and in previous publication [13], the absence of IL10 did not affect the gender difference in the pro-inflammatory response (Figs 1, 2 and 3). Interestingly, the level of anti-inflammatory cytokine, IL10, was also higher in males (Fig 4), thus supporting an overall stronger innate immune response in males. A less robust innate immune response in females may have reproductive importance because better tolerance is needed during pregnancy.

Sex hormones appear to play important roles leading to the higher levels of both pro- and anti-inflammatory cytokines in males. As seen in Figs 1D, 1E, 1F, 2A, 2B, 2C and 4C, young mice did not show the gender difference observed in older mice, and their plasma cytokine levels were mostly between the levels of adult males and females (Figs 1, 2 and 4). The data suggest that both male and female sex hormones contribute to the gender difference in the innate immune response. This conclusion is supported by results from previous surgical models. Using ovariectomized female mice, estrogen treatment was found to suppress acute inflammatory response [36] while in orchiectomized male mice, testosterone treatment augmented endotoxin-induced inflammation [37]. Because the gender difference in the cytokine response can be observed in cultured human mononuclear cells at the mRNA and protein levels [25, 26], sex hormones likely affect the signaling pathway of the innate immune response. Indeed, gender differences in the TLR2 and TLR4 expression were reported [26, 38]. TLR4 expression was found to be higher in endotoxin-challenged human mononuclear white cells from males [26, 38] and in virus-infected mouse splenic lymphocytes from males [26, 38]. Female virus-infected mice, on the other hand, had higher TLR2 expression in the splenic lymphocytes compared to the infected male mice [26, 38].

A gender difference in anorexia, as a part of the sickness response, was observed after the endotoxin exposure (Fig 5 and Table 1). The decrease in food intake was more severe in male mice. Although a direct causal effect was not examined here, more anorexic response in males can be due to the overall higher IL6 and TNFα in males after endotoxin injection (Fig 3) since IL6 and TNFα are known to induce sickness behavior [7]. Gender difference in anorexic response due to inflammation was not examined previously. Because of the similar immune-modulatory roles of sex hormones in mice and humans as discussed above [25, 36, 37], it is possible that in humans, male gender also has a higher risk for inflammation and related anorexic responses.

## Supporting Information

S1 Table Plasma IL6 levels. (XLS)

S2 Table Plasma TNFα levels. (XLS)

S3 Table Plasma IL10 levels. (XLS)

S4 Table Daily food intake. (XLS)

S5 Table Body weight. (XLS)
